# Detection of dengue virus in saliva and urine by real time RT-PCR

**DOI:** 10.1186/1743-422X-7-22

**Published:** 2010-01-27

**Authors:** Telma R Poloni, Anibal S Oliveira, Helda L Alfonso, Larissa R Galvão, Alberto A Amarilla, Dimair F Poloni, Luiz T Figueiredo, Victor H Aquino

**Affiliations:** 1 Departamento de Análises Clínicas, Toxicológicas e Bromatológicas, Faculdade de Ciências Farmacêuticas, Universidade de São Paulo, Ribeirão Preto14040-903, Av. Do Café s/n, São Paulo, Brasil; 2 Centro de Pesquisa em Virologia, Faculdade de Medicina de Ribeirão Preto, Universidade de São Paulo, Ribeirão Preto 14049-900, Av. Bandeirantes 3900, São Paulo, Brasil; 3 Hospital das Clínicas da Faculdade de Medicina de Ribeirão Preto, Universidade de São Paulo, Ribeirão Preto 14049-900, Av. Bandeirantes 3900, São Paulo, Brasil

## Abstract

Early diagnosis of dengue virus (DENV) infection is important for patient management and control of dengue outbreaks. The objective of this study was to analyze the usefulness of urine and saliva samples for early diagnosis of DENV infection by real time RT-PCR. Two febrile patients, who have been attended at the General Hospital of the School of Medicine of Ribeirao Preto, Sao Paulo University were included in the study. Serum, urine and saliva samples collected from both patients were subjected to real time RT-PCR for DENV detection and quantification. Dengue RNA was detected in serum, urine and saliva samples of both patients. Patient 1 was infected with DENV-2 and patient 2 with DENV-3. Data presented in this study suggest that urine and saliva could be used as alternative samples for early diagnosis of dengue virus infection when blood samples are difficult to obtain, e.g., in newborns and patients with hemorrhagic syndromes.

## Finding

Infection with any of the four dengue virus serotypes can lead to a broad clinical spectrum, ranging from sub-clinical infection or an influenza-like disease known as dengue fever (DF) to a severe, sometimes fatal, disease characterized by hemorrhage and plasma leakage that can lead to shock, known as dengue hemorrhagic fever/dengue shock syndrome (DHF/DSS)[1,2]. Laboratory diagnosis of DENV infection is based on virus isolation, detection of virus antigen or RNA and detection of DENV specific antibodies in serum or plasma [3]. Real-time reverse transcription-polymerase chain reaction (RT-PCR) has been developed to detect DENV RNA in human samples [4-7]. This technique is an important tool for the rapid diagnosis of DENV infection allowing early initiation of patient management and specific preventive health measures. The objective of this study was to analyze the usefulness of urine and saliva samples for early diagnosis of DENV infection by real time RT-PCR. The Hospital Ethics Committee of the School of Medicine of Ribeirao Preto, Sao Paulo University has approved this study (Proc. 4921/2007).

**Figure 1:**
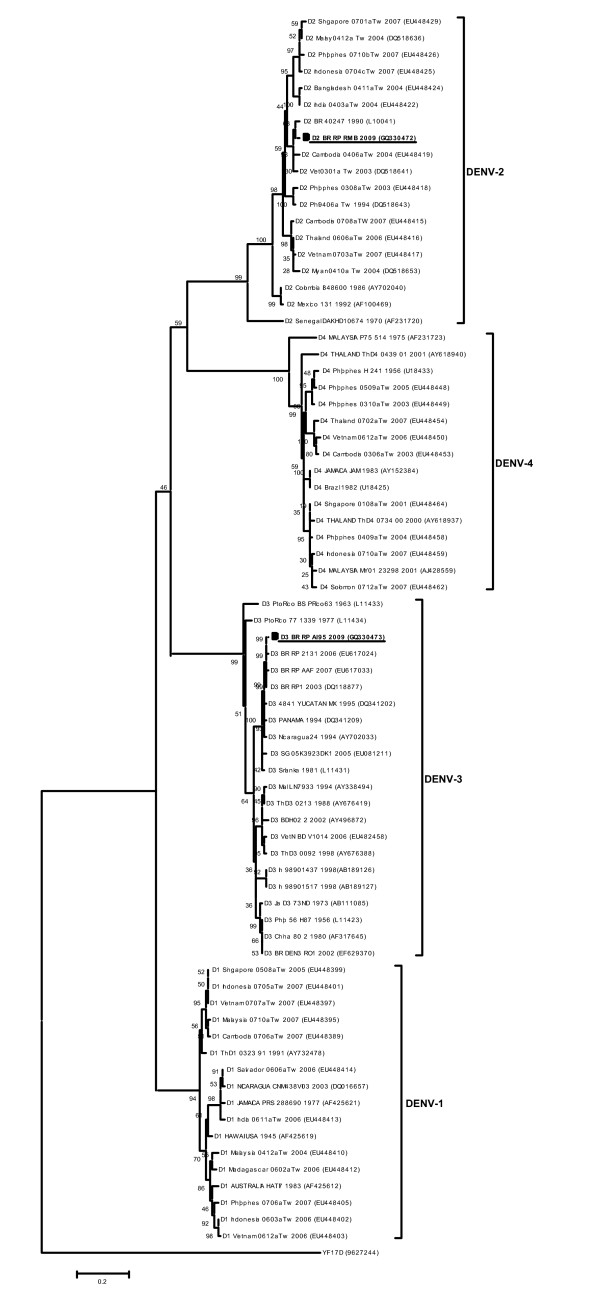
**Phylogenetic tree based on the E gene sequences using Neighbor-joining (NJ) method showing the relationship of viruses isolated in this study with 72 global samples of DENV**. Tamura and Nei (TrN+I+G) nucleotide substitution model was used with a proportion of invariable sites (I) of 0.2096 and gamma distribution (G) of 0.6072 using the hierarchical likelihood ratio test (hLTR). The YF17D (9627244) was used as outgroup. Horizontal branch lengths are drawn to scale. Viruses isolated from patients 1 and 2 are underlined. GenBank accession numbers are indicated in parenthesis.

Two febrile patients attended at the General Hospital of the School of Medicine of Ribeirao Preto, Sao Paulo University, were included in the study. Both patients were female, 30 (patient 1) and 13 (patient 2) years old, and presented symptoms suggestive of DF with high fever, frontal headache, arthralgia, myalgia and retro-ocular pain. The patients received supportive treatment including bedrest, antipyretics, analgesics and oral fluid replacement, and had a good outcome without any complication. Serum, urine and saliva samples were collected, two and nine days after the onset of symptoms, from both patients, and subjected to a quantitative real time RT-PCR for dengue as described earlier [7]. Dengue RNA was detected in all clinical samples collected two days after the onset of symptoms from both febrile patients (Table 1). Serum showed a viral load higher than urine and saliva samples. To identify the virus serotype, the entire E protein gene was amplified from the sera and subjected directly to DNA sequencing for phylogenetic analysis as previously mentioned [8]. This analysis showed that patient 1 was infected with dengue virus type 2 and patient 2 with dengue virus type 3 (Figure 1). The clinical samples were also subjected to a capture ELISA for dengue-specific IgM/IgG as previously mentioned [7]. Both patients presented seroconversion indicating a primary infection (Table 1). For virus isolation, 20 μl of each clinical sample were added to a 25 cm^2 ^flask containing a monolayer of C6/36 cells and seven days after inoculation, the supernatant was subjected to the real time RT-PCR to confirm virus isolation. Dengue virus was isolated from the serum samples collected two days after the onset of symptoms from both patients (Table 1). In order to avoid any kind of contamination, all procedures of virus isolation, RNA purification, protein E gene amplification, PCR product analysis and DNA sequencing were performed in different rooms following good laboratory practice guidelines.

**Table 1:** Dengue diagnostic tests performed in clinical samples from the two patients.

	2 days after onset of symptoms						9 days after onset of symptoms					
	**Patient 1**			**Patient 2**			**Patient 1**			**Patient 2**		

	**Serum**	**Saliva**	**Urine**	**Serum**	**Saliva**	**Urine**	**Serum**	**Saliva**	**Urine**	**Serum**	**Saliva**	**Urine**

Viral load (PFU/ml)	7.9 × 10E2	3 × 10E-1	4 × 10E-1	1.9 × 10E5	1 × 10E-1	5 × 10E-1	N	N	N	N	N	N

IgM*	ND	ND	ND	ND	ND	ND	1.15	0.84	0.60	1.25	0.89	0.54

IgG#	ND	ND	ND	ND	ND	ND	0.78	0.84	0.65	0.82	0.83	0.56

Virus Isolation	DENV-2	N	N	DENV-3	N	N	N	N	N	N	N	N

N: negative. *IgM detection: cut off 0.63; #IgG detection: cut off 0.68. ND: not detectable.

In addition to serum, we were able to detect dengue virus in both urine and saliva samples by real time RT-PCR. In a previous case report study, dengue virus was detected in urine and saliva samples up to 14 days after the onset of fever, when no viremia was detected, suggesting that the virus could remain in urine and saliva for a longer period of time than in serum [9]. However, in our study, dengue virus was detected in serum, urine and saliva samples of both patients, two days after the onset of symptoms, but not after nine days. The lower viral load found in urine and saliva compared to serum suggests that dengue virus would be detected in these clinical samples for a shorter period of time.

Recently, the usefulness of saliva in dengue serologic diagnosis has been studied showing that specific IgM could be detected in patients with primary infection and IgM/IgG in patients with secondary infection [10]. We were able to detect IgM and IgG in the two patients with a primary infection.

Our results suggest that urine and saliva samples could be used for dengue diagnosis in cases where blood samples are difficult to obtain, such as in newborns or in patients with hemorrhagic syndromes. Urine and saliva samples are easy to collect without invasive procedures and urine samples can be collected in large volumes. However, further studies, with a higher number of patients, are needed to confirm these results.

## List of abbreviations

(DENV): Dengue virus; (DF): Dengue fever; (DHF/DSS): Dengue hemorrhagic fever/dengue shock syndrome; (RT-PCR): Reverse transcription-polymerase chain reaction; (NJ): Neighbor-joining; (hLTR): Hierarchical likelihood ratio test.

## Competing interests

The authors declare that they have no competing interests.

## Authors' contributions

TP and AO carried out the real time RT-PCR.

HA and AA carried out the phylogenetic analysis.

LG carried out the serological work.

DP conducted the medical attention.

TP and LF co-wrote and edited the manuscript.

VA organized the overall project and helped edit the manuscript.

All authors read and approved the final manuscript.
